# HiFinger: One-Handed Text Entry Technique for Virtual Environments Based on Touches between Fingers

**DOI:** 10.3390/s19143063

**Published:** 2019-07-11

**Authors:** Haiyan Jiang, Dongdong Weng, Zhenliang Zhang, Feng Chen

**Affiliations:** 1 Beijing Engineering Research Center of Mixed Reality and Advanced Display, School of Optics and Photonics, Beijing Institute of Technology, Beijing 100081, China; 2 AICFVE of Beijing Film Academy, 4 Xitucheng Rd, Haidian, Beijing 100088, China

**Keywords:** text entry, thumb-to-fingers, virtual reality, pressure sensor

## Abstract

We present a text entry technique called HiFinger, which is an eyes-free, one-handed wearable text entry technique for immersive virtual environments by thumb-to-fingers touch. This technique enables users to input text quickly, accurately, and comfortably with the sense of touch and a two-step input mode. It is especially suitable for mobile scenarios where users need to move (such as walking) in virtual environments. Various input signals can be triggered by moving the thumb towards ultra-thin pressure sensors placed on other fingers. After acquiring the comfort range of the touch between the thumb and other fingers, six placement modes for text entry are designed and tested, resulting in an optimal placement mode that leverages six pressure sensors for the text entry and two for the control function. A three-day study is conducted to evaluate the proposed technique, and experimental results show that novices can achieve an average text entry efficiency of 9.82 words per minute (WPM) in virtual environments based on head-mounted displays after a training period of 25 min.

## 1. Introduction

With the development of virtual reality (VR) technology, there are an enormous number of mobile scenarios in virtual environments (VEs) based on head-mounted displays (HMDs) where users need to move by themselves, like running, walking, etc. Meanwhile, inputting a large amount of text is also necessary for these scenarios, such as when performing text recording when operating machines in a three-dimensional maintenance scenario, chatting with friends when walking, and sending short messages to others when playing games. However, the current text entry techniques are not efficient enough for large amounts of text entry in HMD-based virtual environments, especially with eyes free.

One solution for text input during movement is voice input [1,2]. Although voice input has a relatively low error rate in daily conversations, its accuracy may decrease significantly in noisy environments, and its performance on uncommon language such as professional text is not satisfactory either. In addition, mid-air typing [3] and gesture [4] are alternative solutions. The input speed and comfort level of these techniques are relatively low for long-term use, and the latter solution sometimes requires lots of efforts for users to memorize various gestures. Considering the comfortable requirements for text entry in virtual environments where individuals can input a large amount of text while moving, we present HiFinger—a wearable, one-handed text entry technique based on thumb-to-fingers touch—which is implemented by treating the thumb as a stylus to easily touch the widgets on other fingers [5]. Ultra-thin pressure sensors are placed on different parts of fingers and different signals are triggered when the thumb touches these pressure sensors. All input operations can be carried out accurately and quickly with eyes free.

**Figure 1:**
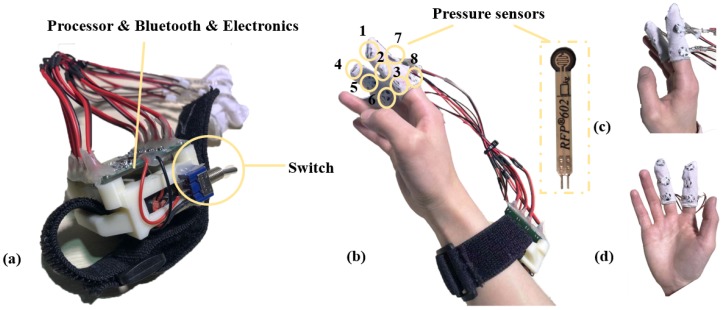
Structure of HiFinger-based interaction system. (**a**) HiFinger with 8 ultra-thin pressure sensors based on the index finger and middle finger; (**b**) one user is wearing the system; (**c**) the side view of the HiFinger-based system; (**d**) the front view of HiFinger-based system.

We propose three factors that need to be considered for large amounts of text entry in mobile scenarios: (1) mobile input; (2) eyes-free input; (3) usability. According to these factors, we designed a two-step input method based on ultra-thin pressure sensors capturing touches by the thumb to the fingers. Six different placement modes of pressure sensors were designed. By synthetic analysis, we found that the mode based on the index finger and the middle finger could achieve the best performance among the six modes. Then, the final placement mode consisting of eight sensors was determined, as shown in Figure 1b. At last, we evaluated the performance of this mode through a three-day, six-trial user study. The experimental results show that after a 250-word training lasting for about 25 min, users can reach an average text entry speed of 9.82 ± 1.12 words per minute (WPM). The technique can also be used together with other wearable devices such as smart watches and augmented reality helmets.

**Figure 2:**
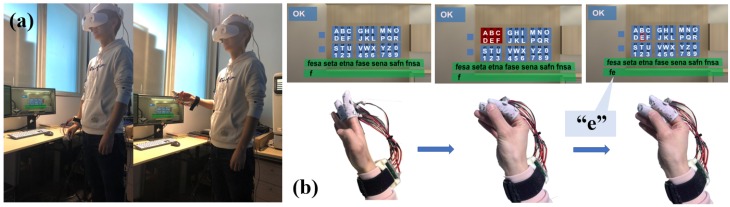
Text entry system based on the HiFinger technique. (**a**) A standing user is inputting with HiFinger in head-mounted display (HMD)-based virtual environments (VEs); (**b**) the process of entering “e” with HiFinger. Step one: a user touches the “Number 1” pressure sensor to specify the “Number 1” character set whose color turns red; step two: a user touches the “Number 5” pressure sensor to specify the character “e”.

We propose the text entry system based on HiFinger (shown in Figure 2). Our contributions are listed as follows:

- We propose a wearable, one-handed, eyes-free text entry technique called HiFinger based on a two-step input mode and touches between fingers, which could be adopted for large-scale text entry tasks.
- We propose six placement modes for HiFinger according to the comfort level of touches between the thumb and other fingers. A user study is further conducted to investigate the performance of each mode to show the advantages and disadvantages.
- We analyze the characteristics of the proposed HiFinger, and a synthetic evaluation is carried out, which proves that the proposed technique is efficient and has good learnability.

The remaining parts of this paper are arranged as follows. Section 2 explores related work in text entry methods. Section 3 introduces the design principles of the text entry techniques in virtual environments, followed by the technical details of the proposed HiFinger technique in Section 4. Then, Section 5 shows the user study of the design of the HiFinger-based interaction system, and Section 6 is the evaluation of the HiFinger-based system. Section 7 discusses some key issues and limitations, and Section 8 concludes this paper.

## 2. Related Work

In this section, related work in text entry in virtual environments is presented at first, and then the text entry techniques for wearable devices are introduced. At last, multi-step input techniques on small devices and finger-based input methods are reviewed.

### 2.1. Text Entry in Virtual Environments

With the maturity of virtual reality VR technology and its widespread applications, a large amount of research has focused on solving input problems in virtual environments. Poupyrev et al. [6] proposed an input system in VEs with a tablet and a pen, using handwriting as a new mode of interaction. But the system could only save the drawn strokes and input simple text and numbers, so the input efficiency was relatively low. Bowman compared the input effects of pinch gloves [7], a one-hand chord keyboard, a soft keyboard using a pen & tablet, and speech. The results showed that speech technology was the fastest, while the least error was generated in the pen and tablet keyboard technology. However, none of these techniques exhibited high levels of performance, availability, and user satisfaction. Meanwhile, the efficiency of speech input [1,2] was sometimes low, and it was prone to discontinuity. Its noise sensitivity and privacy issues [8] also led to its unsuitability for public places. Fels and Hinton [9] used data gloves and neural networks to input words and phrases through gestures. In their system, users needed to remember the meaning of gestures in advance, increasing the difficulty for users.

Many studies have focused on QWERTY keyboard input in VEs and investigated its usability. The controller ray and the hand-based air-click were often adopted to input text in the system with QWERTY virtual keyboards [3,10,11]. Some research focused on virtual keyboard input method on touch screens, and one utilized the user’s muscle memory [12,13]. The physical QWERTY keyboard and the user’s hands are invisible in VEs in HMDs, which may cause input difficulties [14,15], leading to low input accuracy. Meanwhile, presentation methods of the keyboard and hands also affect the input efficiency [16,17,18,19]. QWERTY keyboards is usually only suitable for input with a fixed desktop instead of the input in mobile scenes in HMDs. The ATK [11] system was based on the 3D hand tracking data of the Leap Motion controller, and users could freely input in 3D space using ten fingers. Schick [20] combined handwriting recognition based on the hidden Markov model with multi-camera 3D hand tracking, proposing a vision-based system that could recognize handwriting in mid-air. The recognition rate of characters in this system was 86.15%, but the handwriting input speed was not quick enough for lots of text entry. In the way of air-click or handwriting, the hanging state of hands and the lack of force feedback would reduce the comfort level of users, and the estimation error of the hand position and the recognition error of handwriting were relatively high. The methods were not suitable for efficiently inputting a large amount of text. The technique of touching screens [8,12,16,21] is also an important technique for text input in virtual environments. Since users do not easily feel the position of their hands before pressing for the first time [13], this type of technique may require additional actions to confirm the initial position of the hand.

Head tracking is also a common technique for inputting in VEs. Yu et al. [22] compared three input methods based on the head in VR, in which the entry speed could reach 24.73 WPM by GestureType after a training procedure of 60 min, but it did not support inputting out of dictionary (OOD) words, which brought difficulties for separated characters such as passwords. Marco Speicher [3] compared the input methods of head pointing, controller pointing, controller tapping, freehand, discrete and continuous cursor and found the input using tracked hand-held controllers outperformed all other methods. However, frequent movement of the head may increase motion sickness, so head tracking is not suitable for long-time text entry.

The above methods are not suitable for large-scale text entry of mobile scenes, such as game scenes. Therefore, we need to propose a text entry technique to solve the problem, improving the comfort level of users and the input accuracy, which could ensure that users can easily input a lot of text in VEs.

### 2.2. Text Entry of Wearable Devices

With the popularity of wearable devices, such as HMDs and smart watches, research into wearable input devices has also been addressed.

Some wearable input devices are based on computer vision. Prätorius [23] captured the user’s hand using a wrist-worn camera and infrared laser and reconstructed the hand pose for discrete input with signals arbitrarily mapped to characters or system commands. WatchSense [24] was a wearable device with a depth sensor that users could input in the adjacent and above areas of the skin, but it could only recognize eight types of input. OmniTouch [25] interacted with the surface of the arms, legs, tables, etc., by combining a depth camera and a projector. The user could not use it without eyesight and had to interact with the surface of an object. Sridhar [26] used a camera and chord-like multi-finger gestures to input text in mid-air, which did not require an external target and could be performed without eyes, but this method is likely to cause hand fatigue due to the impending hands, which is not suitable for large-scale text input tasks that take a long time. WatchMe [27] relied on the cloud optical character recognition (OCR) engine to retrieve text from the images captured by the camera of the portable device, which was also difficult to input without eyesight.

Some devices recognize hand motion based on other sensors for input. Wang [28] recognized gestures dynamically using Google’s Soli millimeter wave radar project, achieving a high recognition rate for 11 dynamic gestures (average 87%), but the number of dynamic gestures was limited for text input. Skinput [29] recognized the position of the arm and the finger tapping on the arm and used the skin as the input surface to input by analyzing the mechanical vibration transmitted through the body. Although the input method was convenient to use when users were moving or standing, the recognition accuracy was limited and it was difficult to make accurate input for users under visual occlusion. uTrack [30] used magnetic field (MF) sensing to convert thumb and finger movements into 3D input. It was difficult for users to accurately grasp the angle of finger bending, and the error was large. Airwriting [31] measured hand movements with an accelerometer and a gyroscope attached to the back of the hand, allowing the user to enter text into the computer by writing in the air, but it had a high error rate (11%), and handwriting recognition accuracy was low. SkinTrack [32] consisted of a ring that emitted a continuous high-frequency alternating current signal and an inductive wristband with multiple electrodes for continuous touch tracking on the skin, identifying the 2D position of the touch with an average error of 7.6 mm, resulting in a difficulty of accurate input for users. LightRing [33] measured the 2D position of the fingertip with an infrared sensor and a single-axis gyroscope for input. The device could be used on the thigh or a table, but there were large measurement errors, and the input speed was not high. ThumbText [4] was a thumb-operated text entry approach for a ring-sized touch surface, which allowed for one-handed operation under the eyes-free condition, but the single confirmation of the thumb did not guarantee the user could press the defined area accurately and quickly.

The above techniques are not conducive to accurate and rapid input in the case of visual occlusion. The goal of HiFinger is to provide an eyes-free input technique suitable for mobile scenes.

### 2.3. Multi-Step Text Entry Techniques

The multi-step input technique based on the QWERTY soft keyboard mainly took advantage of users’ familiarity with the layout of the physical keyboard. Swipeboard [34] input characters through two swipes with our spatial memory of QWERTY keyboard. The first slide specified the area where the character was located, and the second slide specified the characters in the area. This technique required the slides’ accurate capture of the user’s finger. SplitBoard [35] displayed half of the QWERTY keyboard at a time, and the user could slide the keyboard display left and right and then confirm the character input. This technique was suitable for small display devices such as smart watches. DriftBoard [36] and ZoomBoard [37] solved the “fat finger problem” on small display devices. DriftBoard allowed the user to type by positioning a movable QWERTY keyboard on an interactive area with a fixed cursor point. ZoomBoard used iterative zooming to enlarge otherwise impossibly tiny keys to a comfortable size to help input.

Many studies have focused on text input using other input devices. PizzaText [38], TwoStick [39], and Sandnes [40] used a hand-held game controller to input text by multiple step confirmation. The entry speed of novices on PizzaText was 8.59 WMP after two hours of training, while that of experts could reach 15.85 WMP. The input speed of TwoStick could be 14.9 WPM after training, while the input speed of Sandnes was 6.75 WPM. This technique required two hands for input, and users had to carry an extra device that was not wearable, which is inconvenient for users. This technique might cause problems such as under- or over-shooting the goal targets [41].

WrisText [42] was a one-handed text entry technique for smartwatches using the joystick-like motion of the wrist. The user input text by rotating the wrist in six directions, with the input speed achieving 9.9 WPM and reaching 15.2 WPM after training. Quikwriting [43] divided letters into groups with base-9 encoding, allowing users to describe entire words or even sentences as a single continuous gesture to input text. Hyeonjoong [44] proposed a text entry technique for wrist-worn watches by determining input letters based on drag direction regardless of touched location. Twiddler [45] was a one-handed chording keyboard device that users could use in eyes-free situations, but users needed to press four buttons for every letter. Compared to input using peripheral equipment, the thumb-to-fingers method can input more accurately by using people’s haptics.

Most of the above techniques are not suitable for mobile scenarios in virtual environments. HiFinger combines pressure sensors with a two-step text input technique to allow fast and accurate text input for HMD-based virtual environments.

### 2.4. Finger-Touch Input

Our text entry technique is a finger-touch input technique, and some research has proposed the use of finger-touch action, including thumb-to-fingers action, for rapid interaction.

The input mode of HiFinger is a little bit like that of the Chording Glove [46], which included three parts: four sensors on the tips of the fingers, three shift buttons on the side of the index finger, and eight function keys on the back of the hand. Users input through a series of chords (combinations of finger presses against the palm and thumb), with entry speeds reaching 8.9 WPM after an 80 min of practice and 16.8 WPM after a 10 h of practice.

As fingers can be an efficient input method, a lot of research has focused on the influence factors. Way [47] evaluated free-hand microgesture usability including finger lifts and finger pinches by using wrist-worn sensor modalities, finding that the lift microgestures (in particular the index lift) were significantly less prone to user error and proposing that microgestures produced relatively more accurate and efficient interactions. WristFlex [48], as an always-available on-body gestural interface, could distinguish subtle finger pinch gestures with high accuracy (>80%) and speed. Thomas [49] proposed a set of pinch-glove-based user interface tools for an outdoor wearable augmented reality computer system and proposed a new glove-based text entry mechanism. N-Fingers [50] allowed the users to control the user interface in an easy, natural way by using the thumb to push “buttons” located in the other fingers, and experimental results showed N-Fingers was a promising interaction technique for wearable computers. DigitSpace [5] addressed two ergonomic factors: hand anatomy and touch precision, when using thumb-to-fingers touch as an input method. Thus, we wanted to take advantage of thumb-to-fingers to design an effective text entry method. Some studies have used thumb-to-fingers as a text input method. H4-TEG [51] used pinches between the thumb and fingers of the users’ right hand with base-4 Huffman code to input text. The character selection was from two to five steps, which was difficult for users to remember. The input speed reached 5.27 WPM after a training of 330 phases and 14.0 WPM through long training. FingerT9 [52] input text leveraging the action of thumb-to-finger touching by mapping a T9 keyboard layout to the finger segments. DigiTouch [53] used thin, partially conductive fabric strips along the fingers and a conductive patch on the thumb pad to conduct the text input by thumb-to-fingers.

Our goal was to achieve efficient text input in HMD-based virtual environments leveraging the thumb-to-finger technique.

## 3. Design Principles

We designed an efficient text entry technique that users could use in mobile scenes, such as walking and standing, for inputting a lot of text according to the following factors.

### 3.1. Mobile Input

There are many scenes where users need to walk, stand, or do other movements in HMDs. Therefore, the proposed technique needs to guarantee that users can use it conveniently in these mobile scenarios. We consider some key factors as follows.

Wearable and portability: It is necessary to ensure that the user can input text in any position. The independent input technique that does not depend on the external environments can ensure the mobility of the input device. Therefore, we designed a new wearable input technique that allows users to easily enter text in any location, eliminating the techniques which could only be used in a fixed location (e.g., physical QWERTY keyboards [14,15,16,17,18,19]). In addition, portability is also one of the factors that need to be considered. Users should easily carry the device, and wearable devices ensure portability. Therefore, products that are inconvenient for users to carry or require help from other people (for example, tablet handwriting input [6] and game controller input [38,39,40]) are not very suitable.

Environmental stability: In mobile scenes, users often face different real environments, including lighting, sound, magnetic field, temperature, and other factors. We need to design input techniques that can adapt to a variety of real environments. For example, in mobile scenes, the lighting environment is unstable everywhere, and the user needs to input accurately and quickly under various lighting conditions. The input method based on computer vision [4,20,23,24,25,27] needs to process the image, and it has a certain degree of light sensitivity and is not suitable for various lighting conditions. Voice input [1,2] will be disturbed by other sounds, so we do not consider it here.

Compatibility: To perform tasks with HMDs, users often need to use other additional devices, such as a controller or a cup. Sometimes users need to perceive and recognize external objects by touch. For example, users drink water with a cup in virtual environments. Therefore, the input technique is not supposed to affect other operations of the user in the virtual environment. During the input process, the one-handed text input method is convenient for the user to use other objects and interact with the scene. Meanwhile, less coverage of hands helps the user to sense the objects better in the real world.

### 3.2. Eyes-Free Input

In scenes where users move, eyes-free input would be beneficial. The biggest feature of the virtual environment generated by HMDs is the invisibility of the user to the surrounding real environment. The user cannot perceive the external environment with vision, which means that the technique cannot rely on the user’s visual perception. Although images or 3D models of real objects can be projected into the virtual environment through registration between the virtual and real objects [8,18,19,54,55], there are still many challenges. Meanwhile, when the user cannot see the input device within the field of view, it may be necessary to turn the head to switch the field of view to perceive the device, which may also cause motion sickness and fatigue [22], and increase the visual search time [56]. We can use an eyes-free text input method to solve these problems. Thumb-to-fingers, which leverages people’s haptics, is beneficial for eyes-free input.

### 3.3. Usability

As this technique needs to be used to input a lot of text, we have to consider the efficiency, learnability, and comfort of this technique, guaranteeing users can input text for a long time.

Efficiency: The Keystrokes per character (KSPC) denotes the number of keystrokes needed, on average, to generate each character of text in a given language using a given text entry technique [57]. The KSPC value will directly affect the text input efficiency. The traditional text input technique for desktop-QWERTY keyboards is the most efficient text input technique. Its KSPC equals 1 without considering the joint input method. As the value of the KSPC increases, the input efficiency decreases. The value of the KSPC in H4-TEG [51] increases from 2 to 5, and the entry time increases from 560 ms to 1400 ms. Decreasing the KPSC is beneficial to increase input speed. In the future, we can also add character correction and prediction algorithms to improve our technique’s input efficiency, which helps to decrease the KPSC.

Learnability: The new input technique should be easy to learn, which is beneficial for users to switch from novices to experts. Efficiency and learnability need to be considered simultaneously to obtain long-time optimal performance [42]. For new input techniques, users need to spend more time and energy on the initial stage of use, and they may not be willing to learn because of frustration [58,59] (for example, gesture input [4,9,28], which requires users to remember a lot of gestures, may reduce the user’s interest in learning). Good input hints and simpler input rules will increase the user’s learning interest and learning speed.

Comfort: The input of a large amount of text requires the user to input for a long time, which requires the adopted input technique to provide a comfortable interaction experience. For example, when users click in the air [20], the hand is suspended, which may cause discomfort in a long task. If the user can place the hand in any position (e.g., vertical suspension, placed in the air, placed on the surface of an object), the fatigue of the hand could be alleviated when users input for a long time. At the same time, good tactile feedback will increase user comfort and improve user performance [60]. Therefore, when we design an input technique, the comfort level of the technique must be considered to ensure its long-time use.

## 4. Technical Details of HiFinger

Taking into account the above factors, we designed a thumb-to-finger one-handed text entry technique based on pressure sensors, which is named HiFinger. The technique is suitable for wearable devices such as HMDs and smart watches, especially for mobile scenarios where users will move (such as walk and stand) and they need to input a large amount of text with visual occlusion.

We attached ultra-thin pressure sensors to different parts of the finger (the front and side of the finger, as shown in Figure 1), so the user can know the positions of these sensors on fingers with eyes-free. They can touch the different pressure sensors quickly and accurately by the thumb to input text. The users can naturally droop the hand, suspend it in the air, or place it on the surface of an object when using this technique, increasing the comfort level when they are engaged in a long task. In addition, as pressure sensors cannot be affected by a variety of lighting environments, it can be adapted to a variety of real environments where HMDs are used, no matter how bright or dark the environment is. As text entry needs to input at least 26 letters and a two-step input method is selected here, HiFinger needed at least six input points for text entry. Finally, we used 8 RFP-602 pressure sensors (Figure 1, measurement accuracy: 0.01 N, range 1–200 N, diameter: 10 mm, thickness: 0.2 mm), which are fixed on the index finger and the middle finger with the help of two elastic finger sleeves. In the future, we will consider attaching each sensor to a finger ring for users with different sizes of hands, which would make the device more convenient to use. These sensors connect the data processing unit via a wire, and the data processing unit transmits the touch data to the computer via Bluetooth after converting the touch pressure analog signal to a digital signal. Figure 1 shows the hardware of this technique based on the index finger and the middle finger.

**Figure 3:**
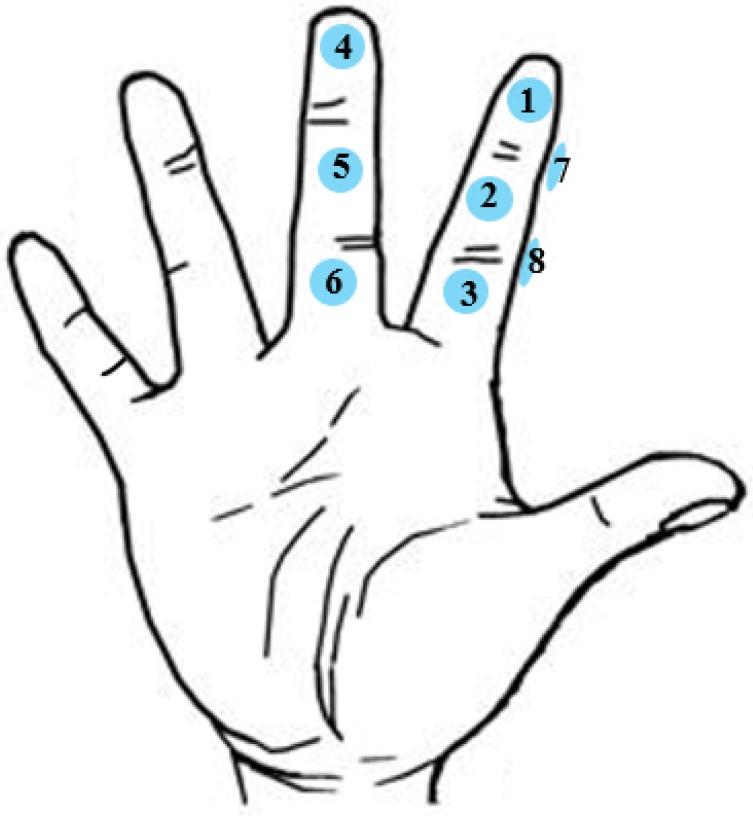
The pressure sensor deployment of HiFinger.

**Figure 4:**
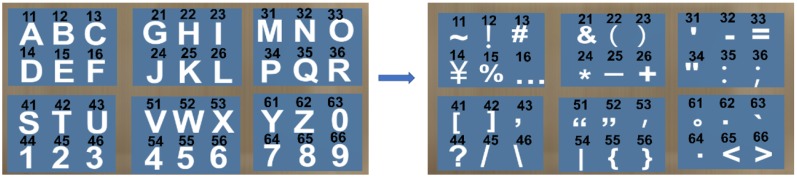
The switch between the alphabet and numbers and specific symbols.

**Figure 5:**
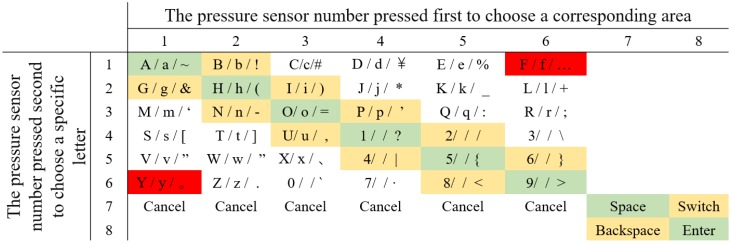
The corresponding serial number of inputting letters.

We assign the pressure sensors numbers 1 to 8 according to their position on the fingers (Figure 3). The pressure sensors No. 1 to 6 are used for the entry of characters and numbers or special symbols, while the pressure sensors No. 7 and No. 8 are used as function keys for the input of “Space”, “Enter”, “Backspace”, and the switch among uppercase letters, lowercase letters, and special symbols (Figure 4). Our technology is visualized by displaying the letter layout in HMDs (Figure 4 left), which is set to six areas according to the number of pressure sensors, each of which has six characters. This configuration offers good consistency for both the first and the second touches. We assign each character a serial number according to the letter layout. For example, the number of “A” is 11, the number of “B” is 12, and the number of “Z” is 62. The user needs to remember the serial number of each sensor or input according to the letter layout displayed by HMDs and then touch the sensor corresponding to the serial number to input characters. For example, if a user wants to input “e” and the serial number of “e” is 15, the user first needs to touch the No. 1 pressure sensor on the index finger and then touch the No. 5 pressure sensor on the middle finger. Figure 2b shows the process of entering the character “e”. When the user makes the first touch (i.e., the No. 1 pressure sensor), the area including the selected character is determined, and the background of the area becomes red as a hint. Afterwards, when the user makes the second touch (i.e., the No. 5 pressure sensor), the character “e” is selected, and the background color of this area turns to blue again. Figure 5 illustrates the corresponding serial number of inputting letters, where “Cancel” is used to cancel the corresponding selection area for the first time of touching. “Backspace” is used to delete the text that has been input, and “Switch” is used to switch letters between uppercase letters, lowercase letters, and special symbols. Blank areas can be defined for the input of other letters, but we have not defined these here. The input letters in the green area are touched on the same sensor in two steps, meaning the input time can be minimal, while the letters in the yellow area are touched on the adjacent area, so the input time will increase, relatively. The distance between the two sensors in the red area is the farthest, and the input time will be maximal. In the future, the letter layout can be shrunk and placed at the non-center of the screen or directly hidden, and the text entry can rely on the user’s memory and muscle memory. To avoid false touches, we set the pressure perception threshold for each sensor; a touch is considered only if the pressure detected by the sensor exceeds the threshold.

The response speed accuracy of the RFP-602 pressure sensor is a microsecond. We assume that the time for each touch is 280 ms according to the assumption of Bajer et al. [51], including 90 ms for the finger and thumb to meet, 100 ms of contact time, and 90 ms for separation. Based on this assumption, the input speed with HiFinger could reach 21.43 WPM, which is higher than H4-TEG (18.1 WPM), because the KPSC of HiFinger is equal to 2 and the KPSC of H4-TEG [51] is greater than or equal to 2, and even up to 5. Thus, we believed that the proposed HiFinger would perform well. We conducted experiments to investigate its performance.

## 5. User Study 1: HiFinger Design

The ultimate goal of the technique is that the user can input at least 29 characters, including “Space”, “Backspace”, and “Enter”, to ensure that the user can input normally. We use a two-step input method to improve user input efficiency and, in this mode, at least 6 input signals are required. In the first experiment, we investigated the comfort level of the user using the thumb to touch different parts of different fingers, including the front and sides of the fingers. Based on the results, we designed six different pressure sensor placement modes and conducted a user study in the second experiment, exploring efficient solutions of this technique.

### 5.1. Experiment 1: Comfort Zone Investigation

Users have different levels of comfort when using the thumb to touch different parts of other fingers. When users input text continuously, the touch time is long, which requires that users have a high level of comfort using this technique. Therefore, we adopted a 7-point semantic opposites scale to investigate the level of comfort when users use the thumb to touch other parts of the hand (1—the most uncomfortable, 7—the most comfortable).

#### 5.1.1. Participants

Thirteen participants (5 females and 8 males) between the ages of 21 and 35 (M = 28) were recruited from our university to take part in this study. All of them were right-handed.

#### 5.1.2. Experiment Design and Procedure

**Figure 6:**
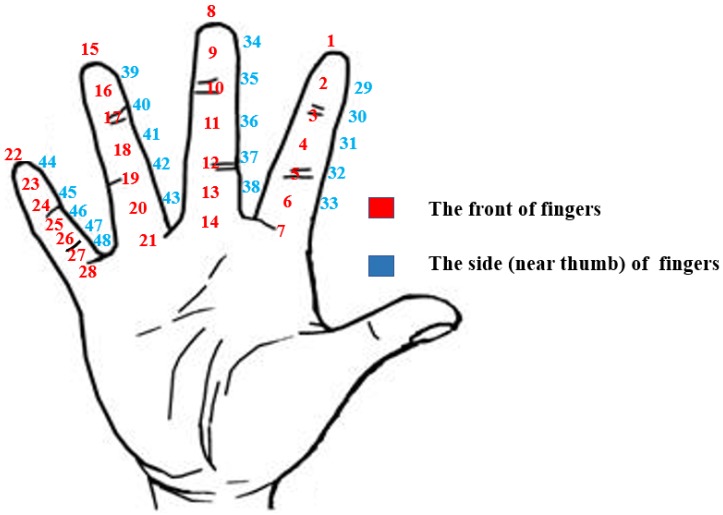
The serial number of different areas of the fingers.

The study lasted about 10 min per participant. We divided the surface of fingers into a total of 48 areas according to the knuckles, the front, and the side surface of the fingers. As shown in Figure 6, on the palm’s surface, each finger was divided into the fingertip area, the first joint area, the second joint area, a third joint area, the area between the fingertip and the first joint, the area between the first joint and the second joint, and the area between the second joint and the third joint. There were 7 points on the front surface of each finger and a total of 28 points on the 4 fingers. On the side surface (near the thumb) of the finger, each finger was divided into the first joint area, the second joint area, the area above the first joint, the area between the first joint and the second joint, and the area below the second joint. There were 5 points on the side surface of each finger and a total of 20 points on the 4 fingers. Each participant touched the 48 different areas using their thumb and scored according to the level of comfort.

#### 5.1.3. Results

**Figure 7:**
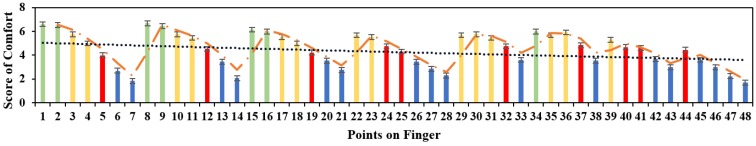
The scores of the level of comfort of the different areas of hand.

A general linear model (GLM) repeated measures analysis of variance (ANOVA), with Greenhouse-Geisser correction or Huynh-Feldt correction, was conducted in this experiment. Figure 7 shows the scores of the level of comfort of the different areas thumb touching. The GLM Repeated Measures ANOVA results indicated there was a significant difference of the comfortable degree of the all points on the fingers $\left(F_{4.852,58.231}=19.657,p<0.001\right)$. Polynomial contrasts demonstrated that there was a significant linear trend $\left(F_{1,12}=16.392,p=0.02\right)$.

There were significant differences of the comfortable degree of the 7 different points of the front surface of each finger—Index finger $\left(F_{2.949,35.388}=65.587,p<0.001\right)$, middle finger $(F_{3.161,37.931}=58.501$, p<0.001), ring finger $\left(F_{2.450,29.398}=25.988,p<0.001\right)$, little finger $\left(F_{6,72}=26.202,p<0.001\right)$. And there all were significant linear trend for 7 points of the front surface of each finger—Index finger $(F_{1,12}=251.597,$p<0.001), middle finger $\left(F_{1,12}=274.000,p<0.001\right)$, ring finger $\left(F_{1,12}=52.673,p<0.001\right)$, little finger $\left(F_{1,12}=65.135,p<0.001\right)$.

There were significant differences of the comfortable degree of the 5 different points of the side surface (near thumb) of each finger—Index finger $\left(F_{4,48}=12.638,p<0.001\right)$, middle finger $\left(F_{4,48}=58.501,p<0.001\right)$, ring finger $\left(F_{2.625,27.174}=16.587,p<0.001\right)$, little finger $\left(F_{1.920,32.618}=19.474,p<0.001\right)$. And there all were significant linear trend for 5 points of the side surface of each finger—Index finger $\left(F_{1,12}=30.094,p<0.001\right)$, middle finger $\left(F_{1,12}=35.981,p<0.001\right)$, ring finger $\left(F_{1,12}=26.294,p<0.001\right)$, little finger $\left(F_{1,12}=31.827,p<0.001\right)$.

#### 5.1.4. Discussion

**Figure 8:**
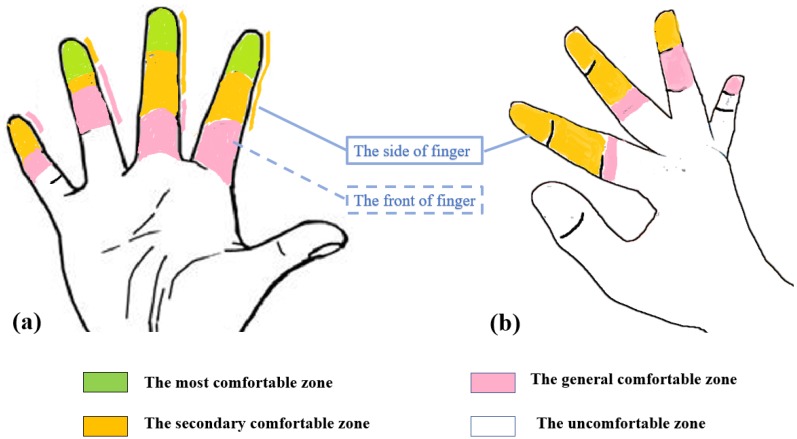
Zones of the different level of comfort on right hand. (**a**) The heat map of the front of fingers; (**b**) The heat map of the side of fingers.

We defined the areas where the average score over 6 as the most comfortable zone (number 1, 2, 8, 9, 15, 16, 34, i.e., the green bars), the average score between 5 and 6 as the secondary comfort zone (yellow bars), and the average scores between 4 and 5 as the general comfort zone (red bars). And the three type areas where the average score over 4 are defined as comfort zones, and the others areas as uncomfortable zone (blue bars). The linear trend indicated that the farther the finger was from the thumb, the lower the level of comfort is, and the farther the zone of the front or the side of each finger was from the fingertip, the lower the level of comfort is. In addition, the level of comfort of the side surface of each finger is lower than the level of comfort of the adjacent front surface. We generated a heat map based on the level of comfort, where Figure 8a displays the heat map of the front of fingers and Figure 8b displays the heat map of the side of fingers. Green areas represent the most comfortable zone; yellow areas represent the secondary comfortable zone; red areas represent the general comfort zone; white areas represent the uncomfortable zone. According to the comfortable level of different zones of fingers, we can design the placement modes of pressure sensors. When the technique is used for a long time, the level of comfort of touch will affect the text entry speed and accuracy.

### 5.2. Experiment 2: Placement Modes Comparison

**Figure 9:**
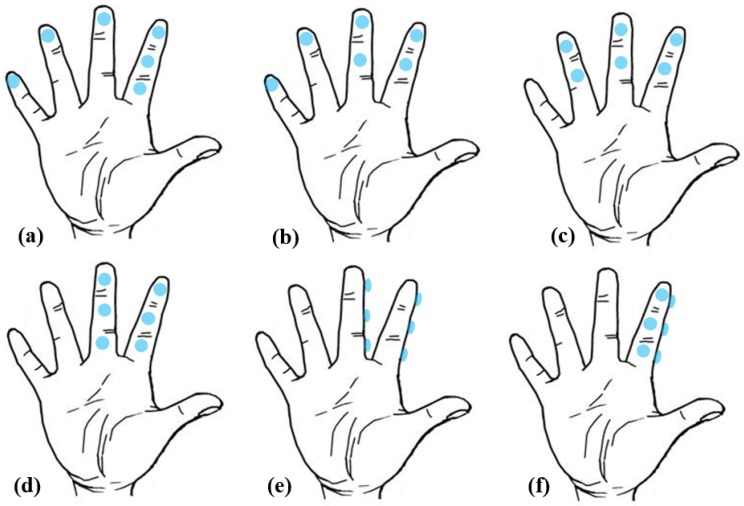
The different placement modes of pressure sensors. (**a**) Type 1; (**b**) Type 2; (**c**) Type 3; (**d**) Type 4; (**e**) Type 5; (**f**) Type 6.

The distance between each sensor and the thumb and the distance between the various sensors will affect the workload and input speed. Based on the results of Experiment 1 and Fitt’s law [56], we designed six different possible placement modes of pressure sensors for HiFinger, as shown in Figure 9. Six pressure sensors were placed in the comfort zones. Type 1: Place 4 pressure sensors on the four fingertips and 2 sensors at the side of the index finger (Figure 9a). Type 2: Place 4 pressure sensors on the four fingertips and 2 sensors on the front of the index finger and the middle finger, respectively (Figure 9b). Type 3: Place 3 pressure sensors on the fingertips of the index finger, middle finger, ring finger and 3 sensors on the front of the three fingers, respectively (Figure 9c). Type 4: Place 2 pressure sensors on the fingertips of the index and middle fingers and the left 4 pressure sensors on the front of the two fingers as shown in Figure 9d. Type 5: it was similar with Type 4, but all 6 sensors were placed on the side surface of the index finger and the middle finger, as shown in Figure 9e; Type 6: Place all 6 sensors on the index finger with 3 placed in the front surface and 3 placed on the side surface, as shown in Figure 9f. We wanted to study the impact of placement modes on input performance.

#### 5.2.1. Hypotheses

- H.1. The input speed of Type 4, Type 5 and Type 6 will be higher than Type 1, Type 2 and Type 3. According to Fitt’s law, we guess the shorter the distance between the two sensors is, the faster the input speed is. Similarly, the shorter the distance between each sensor and the thumb is, the faster the input speed is.
- H.2. The input speed of Type 4 will be higher than Type 5. Although they have a similar arrangement, when users want to touch the sensors on side of the middle finger in Type 5, more movement for the index finger will be needed, as well as the level of touching comfort of the side of the finger is lower than the front of the finger. So the input speed of Type 5 will be lower.
- H.3. The input speed of Type 4 will be higher than Type 6. The thumb in Type 6 needs to move on two vertical faces, which may increase the movement time and decrease the level of comfort. So the input speed of Type 6 will be lower.
- H.4. Workload: Type 1 > Type 2 > Type 3 > Type 5 > type 6 > Type 4. Since the thumb and other fingers move less, the workload will be smaller.

#### 5.2.2. Participants

Fourteen participants (3 females and 11 males) with the age ranging from 20 to 30 (M = 23) were recruited from our university to take part in this experiment. According to the pre-experiments questionnaire, 5 participants had limited experience with VR, while 9 participants had abundant experience with VR. And they all were right-handed and have no experience with touch-sensitive devices.

#### 5.2.3. Apparatus

**Figure 10:**
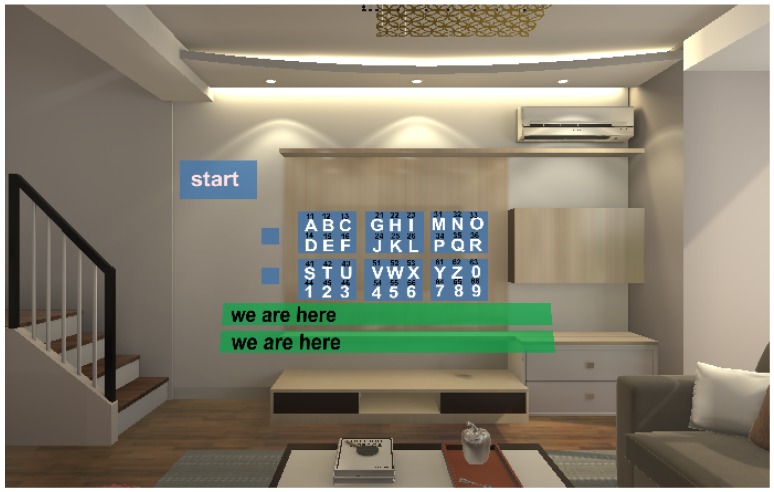
The part of experimental scenario in the HMD.

The experiment was conducted on a desktop computer with the Intel Core i7 processor and the NVIDIA GTX 1080Ti graphics card, and the software was implemented with C# in Unity 2018.2.0.f2. Our virtual environments were displayed in the DELL Visor Headset VR118 that allowed participants to be completely immersed into the virtual world. Figure 10 shows the part of experimental scenario in the HMD, where the letter layout was placed in the center of field of view and its distance to the user is about 3 m. It takes about 30 degrees of horizontal view.

#### 5.2.4. Experimental Design and Procedure

The task used a repeated-measure within-participant design with Modes (6 different placement modes of pressure sensors) as an independent variable. To avoid the effect of the sequence, the ordering of modes that every participant adopted was random.

This experiment lasted about 70 mins for every participant. The task was divided into 6 blocks: one for each modes. Each block consisted 4 sequences of trials. In each sequence, participants had to input 36 characters displayed in the VR environment, including all the letters, some numbers, the Space-key and the Backspace-Key. The characters were input according to the code sequence, preventing participants from spending too much time to find the serial number of characters. In this way, we could simulate the performance when users are familiar with every mode. This could help us find the best input method among the six. Because there is no training phase before the experiment, we regarded the first sequences entry as training processes. In this experiment, we collected 6 (types) × 3 (repetitions) × 15 (participants) = 270 sequences of trials.

After every block, participants needed to complete the questionnaire including the NASA Task Load Index (NASA-TLX) questionnaire [61] and three questions about the likeability, familiarity and the level of comfort of each mode. Likeability was used to measure how much participants liked this type mode they used; familiarity was used to measure how much participants were familiar with the type mode after they used; the level of comfort was used to measure the degree of comfort that participants felt after they used this type mode. We adopted a 5-point Likert scale in the three-question questionnaire. Participants sat to complete the experiments, which typing condition was same as the standing condition where users hand could be placed anywhere.

#### 5.2.5. Results

The main dependent measures for the task were the entry speed, the error rate, and the workload. We measured the entry speed in words per minute (WPM) [62] using the following formula:

(1) $$WPM=\frac{\left|S\right|}{T}\times 60\times \frac{1}{5},$$

where *S* is the length of the transcribed string, and *T* is the task completion time in seconds. In this experiment, we measured the uncorrected errors as the error rate, which did not allow for the correction of characters because correcting mistakes would cost participants more time. The increase in accuracy would affect entry speed.

A GLM repeated measures ANOVA with Greenhouse–Geisser correction or Huynh–Feldt correction was conducted in this experiment. A paired T-test was performed to show the difference between two blocks. In this experiment, we saw the first sequence data as the training data and analyzed the latter 3 sequences.

#### Entry Speed

The GLM repeated measures ANOVA results indicated that there was a significant difference in the text entry speed of three sequences of all different types $\left(F_{5,65}=2.459,p<0.05\right)$, but there was not a significant difference among Type 4, Type 5, and Type 6 $\left(F_{2,26}=0.40,p=0.961\right)$. The paired T-test results indicated that there was no significant difference between Type 4 and Type 5 (t(13)=0.393,p=0.701), Type 5 and Type 6 (t(13)=0.087,p=0.932), or Type 4 and Type 6 (t(13)=0.161,p=0.875).

**Figure 11:**
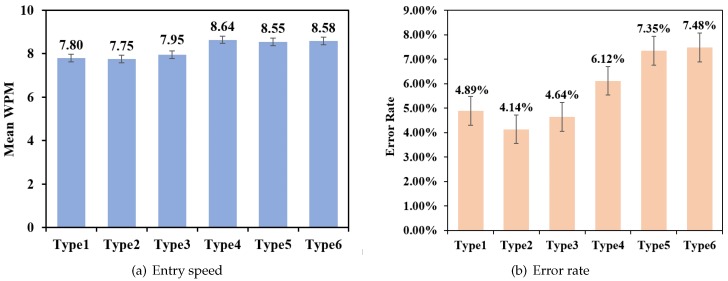
Mean text entry speed (**a**) and mean error rate (**b**) of 6 different placement modes.

Figure 11a shows the average text entry speed of three sequences for 6 different modes. Overall, the average values of the text entry speed for Type 1, Type 2, Type 3, Type 4, Type 5, and Type 6 were 7.80±1.48 WPM, 7.75±2.02 WPM, 7.95±1.54 WPM, 8.64±1.86 WPM, 8.55±1.68 WPM, and 8.58±1.71 WPM, respectively, for novices.

**Figure 12:**
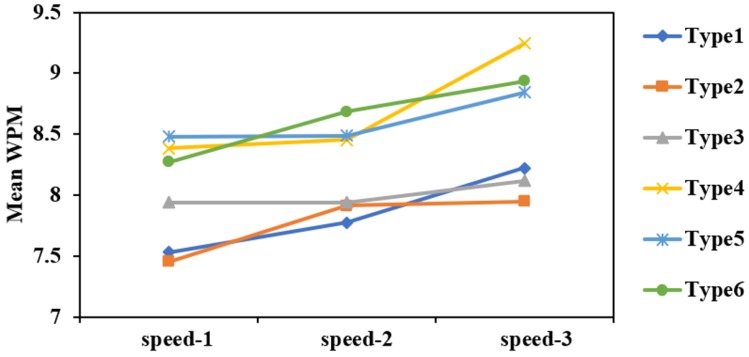
The trend of the entry speed for 6 different placement modes. WPM—words per minute.

For each type, there was a rising trend in the entry speed (Figure 12). Speed-1 refers to the entry speed of the second sequence, since we regarded the first sequence as training data; speed-2 refers to the entry speed of the third sequence; speed-3 refers to the entry speed of the fourth sequence. The GLM repeated measures ANOVA results indicated that there was a significant difference in entry speed for all types except Type 3 and Type 5: Type 1 $\left(F_{2,28}=2.459,p<0.001\right)$, Type 2 $\left(F_{2,28}=7.633,p<0.05\right)$, Type 3 $\left(F_{2,28}=0.343,p=0.713\right)$, Type 4 $\left(F_{2,28}=11.538,p<0.001\right)$, Type 5 $\left(F_{2,28}=0.946,p=0.401\right)$, Type 6 $\left(F_{2,28}=4.310,p<0.05\right)$. Polynomial contrasts demonstrated that they all have a significant linear trend, except Type 3 and Type 5: Type 1 $\left(F_{1,14}=8.7554,p<0.05\right)$, Type 2 $\left(F_{1,14}=9.675,p<0.05\right)$, Type 3 $\left(F_{1,14}=0.541,p=0.474\right)$, Type 4 $\left(F_{2,28}=27.407,p<0.001\right)$, Type 5 $\left(F_{1,14}=1.365,p=0.262\right)$, Type 6 $\left(F_{1,14}=5.894,p<0.05\right)$.

#### Error Rate

The GLM repeated measures ANOVA results indicated that there was no significant difference in error rate for all 6 types $\left(F_{5,65}=1.817,p=0.112\right)$, but there was a significant linear trend $\left(F_{1,13}=6.070,p<0.05\right)$. Figure 11b shows the error rate across 6 different placement modes. The paired T-test results showed that there was no significant difference between any two types, except Type 2 and Type 6 (t(13)=-2.438,p<0.05) and Type 2 and Type 5 (t(13)=-2.204,p<0.05).

#### Workload, Likability, Familiarity, and Comfort

**Figure 13:**
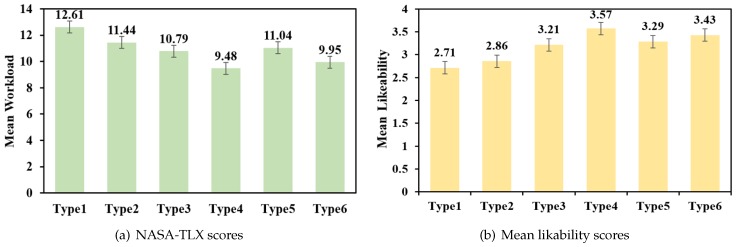
The NASA-TLX workload scores (**a**) and mean likability scores (**b**) for 6 different placement modes.

Figure 13a shows the NASA-TLX workload scores (the lower, the better) across 6 different placement modes. Repeated measures ANOVA results indicated that there was a significant difference in the workload of the 6 types $\left(F_{5,65}=3.273,p<0.05\right)$, with Type 4 requiring a lower workload than other types. There was also a significant linear trend $\left(F_{1,13}=7.985,p<0.05\right)$. The paired T-test revealed that there was similar workload between Type 4 and Type 6 (t(13)=0.428,p=0.676). Figure 13b illustrates the mean likability scores across the 6 different placement modes. Type 4 had a relatively higher score, but there is no significant difference of 6 types $\left(F_{5,65}=1.821,p=0.121\right)$.

**Figure 14:**
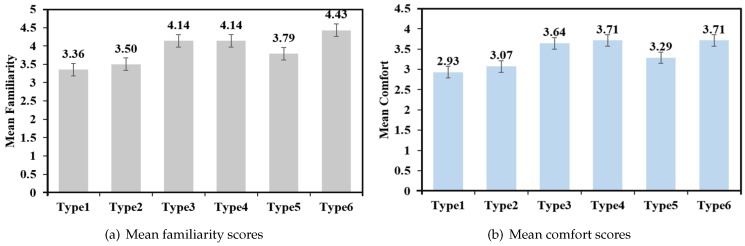
Mean familiarity scores (**a**) and mean comfort scores (**b**) of 6 different placement modes.

Figure 14a shows the mean familiarity scores across the 6 different placement modes. Type 6 had a relatively higher score, and the GLM repeated measures ANOVA indicated that there was a significant difference across the 6 different placement modes $\left(F_{5,65}=3.275,p<0.05\right)$. There was no significant difference between Type 4 and Type 6 (t(13)=1.170,p=0.263). Figure 14b shows the mean comfort scores across the 6 different placement modes. Type 4 and Type 6 had relatively higher scores, but there was no significant difference $\left(F_{5,65}=2.121,p=0.074\right)$.

#### 5.2.6. Discussion

The study results offer evidence to support our hypotheses. By decreasing the distance between the thumb and the pressure sensors and the distance between one pressure sensor and another, the workload declined (H.4.) and the entry speed increased (H.1.). In addition, the range of the fingers’ motion also affected the accuracy of the input (H.2. and H.3.). Based on our experimental results, the mode with the sensor placed on the front of the index finger and middle finger, in Type 4, has the greatest potential for practical use, and we need more investigations to evaluate its performance.

## 6. User Study 2: Performance Evaluation

We conducted a three-day user study including six trials to evaluate the performance of the mode based on Type 4. Since Type 6 also had a higher performance, we considered improving the usability of Type 4 by adding two function keys on the side surface of the index finger, as shown in Figure 1, expanding the function of this input mode. This design would enable the input of all letters, numbers, symbols, and function keys, like “space”.

We adopted the protocol from [4] to simulate expert performance. According to the serial number of letters, we chose 6 letters—“a”, “s”, “t”, “e”, “n”, “f”—that had a high frequency of use in words and different distances between the two touches, such as “a” needing a double touch on the same press sensor and “f” having the largest distance from “No. 1” sensor to “No. 6” sensor, leading to 100 4-letter words such as “east” or “step”.

### 6.1. Participants, Apparatus, and Materials

Nine participants (3 females and 6 males) with ages ranging from 20 to 30 (M = 24) years old were recruited for this study. All of them were right-handed. We used the same apparatus and devices as shown in Experiment 2.

### 6.2. Experiment Design and Procedure

A repeated measure within-participant design with blocks (1 to 6) as an independent variable was used in this study. The experiment consisted of 6 blocks separated into 3 days, and every day consisted of 2 blocks separated by at least 5 h. A block consisted of 6 sets (one is composed of 7 words) and each word was randomly picked from the list of 100 words that were generated with these 6 letters. In this experiment, we collected 6 blocks × 6 sets × 7 words = 252 words per participant, for a total of 2268 acquisitions.

Prior to the experiment, there was no practice as we wanted to evaluate the learning process. In the experiment, participants needed to correct the error as we wanted to simulate real entry. They were sitting during the experiment.

### 6.3. Results

We measured the entry speed by entered words per minute and the error rate. The error rate was calculated according to the formula below:

(2) $$Error\;Rate=\frac{unnoticed\;errors+corrected\;errors}{unnoticed\;errors+corrected\;errors+correct}\times 100\%.$$

#### 6.3.1. Entry Speed

**Figure 15:**
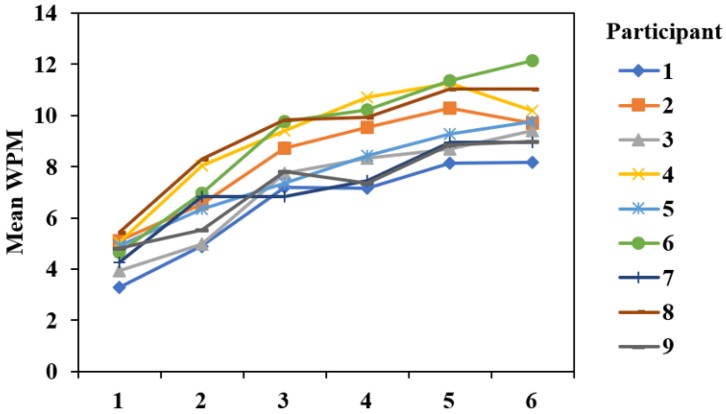
Entry speed of every participant across the 6 blocks.

**Figure 16:**
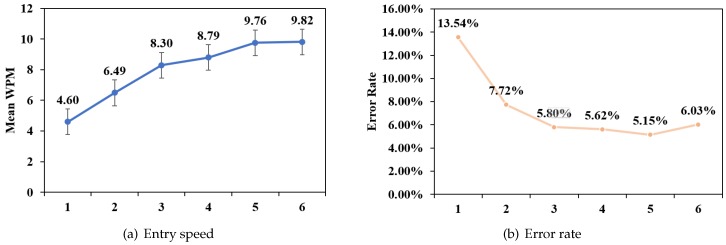
Mean entry speed (**a**) and error rate (**b**) across the 6 blocks.

Figure 15 shows the entry speed of every participant, and Figure 16a shows the average entry speed of all participants across the 6 blocks. The GLM repeated measures ANOVA results indicated that there was a significant difference in the text entry speed of the 6 blocks $\left(F_{5,40}=122.811,p<0.001\right)$, and polynomial contrasts demonstrated that there was a significant linear trend $\left(F_{1,8}=304.803,p<0.001\right)$. In the last block, entry speed achieved 9.82±1.12 WPM.

#### 6.3.2. Error Rate

Figure 16b shows the error rate across the 6 blocks. The GLM repeated measures ANOVA results indicated that there was a significant difference in the error rate of the 6 blocks $\left(F_{5,40}=7.774,p<0.001\right)$, and polynomial contrasts demonstrated that there was a significant linear trend$\left(F_{1,8}=14.987,p<0.05\right)$. But there was no significant difference among 3 to 6 block. After the second block, the increase in entry speed did not significantly influence the error rate.

## 7. Discussion and Future Work

We present the insights we learned from this work, discuss the limitations, and propose future work.

### 7.1. Usability

In our work, we conducted experiments where participants sat to input text. In the future, we will explore the usability of this text entry technique in different scenarios, for example, where users are standing, walking, or even running. In our work, we only conducted the experiments with the English alphabet, and in the future, more studies of text entry in other languages, such as Chinese and Japanese, are needed. In addition, the entry efficiency of different amounts of text using HiFinger, such as passwords, short dialogues, and a long paragraphs of text, requires more study.

### 7.2. Design of Placement Modes of Sensors

This work is our first attempt to design a one-handed text entry technique for some virtual environments based on HMDs where users could move, such as walking or standing. It can also be used when users sit down or in other stationary situations. At present, the performance of a placement mode based on the middle finger and the index finger is better. In this placement mode, we fixed sensors in fixed positions. In the future, sensors can be fixed according to the size of each hand. For example, each sensor can be fixed on a ring, and users can adjust the positions of each ring according to the size of the hand. We have not conducted research on non-dominant hands or on both hands. The occupation of non-dominant hands will release our dominant hand, to some extent, ensuring that our dominant hand can easily perform other operations. Two-step touches with both hands for input also requires more exploration.

### 7.3. Layout of Characters in Virtual Environments

In this study, we explored this input technique using a single character display layout. The display layout of characters will also affect the input efficiency. We will investigate the modification of the layout of characters as a long-term research project, to continuously improve the design of the layout. For example, in this study, we observed that when the two touches for a character are at the same location, the entry speed is significantly faster than when the two touches are at two locations far from each other. In our daily language, some characters have a high frequency of use, such as “e” and “t”, and some characters have a lower frequency of use, such as “z” and “x”, according to which we can design more appropriate display layouts and text entry rules. In addition, we conducted the experiments with the letter layout in the center of the field of view. We will conduct additional experiments to verify this text entry technique can be used when the letter layout is small and placed at the edges of the field of view or even hidden.

### 7.4. Function Expansion

In this study, we used the detected signal to input text. We can also use these input signals as control buttons for different interactions, like the work of Bowman [62]. For example, it can be used for menu control, navigation control, etc. Meanwhile, different placement modes of sensors are suitable for different interactions, which is an advantage of fixing the sensors separately (for example, each sensor is fixed on a ring), helping us to use the same device for more interactions.

### 7.5. Input Method Expansion

Currently, we use the thumb to meet other fingers for input. We can also use the signal from the touch signal between the sensor located on the fingertip and other objects to input text. As we all know, HMDs have various scenes in which users can sit still, walk, and stand. For example, when sitting down, the user can tap the desktop or other surface to input text. Even the thigh can also be used as an input surface. When standing, the user can input text by the touch between the thumb and fingers or the fingertips and other parts of the body. In the future, there will be more different states (standing, walking, sitting) for users in VEs, and they would need to constantly switch. Therefore, the design of a switchable input mode for such an application will help users input more efficiently in different states.

## 8. Conclusions

In this paper, we introduced HiFinger, a one-handed text entry technique based on pressure sensors and the two-step input method. This technique allows users to input text in VEs eyes-free, especially in the mobile scenarios where users need to move, such as walking and standing. Haptics, wearability, and comfort make this technique easy to use and suitable for a large amount of text entry. After exploration, according to the requirements (mobile input, eyes-free input, and usability) of the input device in virtual mobile scenarios, we designed a text entry technique based on the two-step input method. By conducting two experiments, the placement mode of the technique was determined, and then we evaluated the usability and learnability of HiFinger. The experimental results show that after a discrete training period of about 25 min, the entry speed of novices can achieve 9.82 WPM. We believe that HiFinger is efficient and easy to use, and more training will help to improve the users’ performance. This research is the first exploration of this technique. There are many things that could be improved and more exploration in the future will help us improve the efficiency and usability of this technique. With the popularity of VR applications, there will be more mobile scenarios where users need to change body states (such as walking) and input text, and eyes-free input and wearability of the input technique are the basis for these applications. HiFinger could be an important foundation for this type input technique in the future.

## References

[1] Bowman D.A., Rhoton C.J., Pinho M.S. (2002). Text Input Techniques for Immersive Virtual Environments: An Empirical Comparison. Proc. Hum. Factors Ergon. Soc. Annu. Meet..

[2] Lyons K., Skeels C., Starner T., Snoeck C.M., Wong B.A., Ashbrook D. Augmenting conversations using dual-purpose speech. Proceedings of the 17th Annual ACM Symposium on User Interface Software and Technology.

[3] Speicher M., Feit A.M., Ziegler P., Krüger A. Selection-based Text Entry in Virtual Reality. Proceedings of the 2018 CHI Conference on Human Factors in Computing Systems.

[4] Kim J., Delamare W., Irani P. ThumbText: Text Entry for Wearable Devices Using a Miniature Ring. Proceedings of Graphics Interface.

[5] Huang D.Y., Chan L., Yang S., Wang F., Liang R.H., Yang D.N., Hung Y.P., Chen B.Y. DigitSpace: Designing Thumb-to-Fingers Touch Interfaces for One-Handed and Eyes-Free Interactions. Proceedings of the 2016 CHI Conference on Human Factors in Computing Systems.

[6] Poupyrev I., Tomokazu N., Weghorst S. Virtual Notepad: Handwriting in immersive VR. Proceedings of the IEEE 1998 Virtual Reality Annual International Symposium (Cat. No. 98CB36180).

[7] Bowman D.A., Wingrave C.A., Campbell J., Ly V., Rhoton C. (2002). Novel uses of Pinch Gloves™ for virtual environment interaction techniques. Virtual Real..

[8] Grubert J., Witzani L., Ofek E., Pahud M., Kranz M., Kristensson P.O. Text entry in immersive head-mounted display-based virtual reality using standard keyboards. Proceedings of the IEEE Conference on Virtual Reality and 3D User Interfaces (VR).

[9] Fels S.S., Hinton G.E. (1998). Glove-TalkII-a neural-network interface which maps gestures to parallel formant speech synthesizer controls. IEEE Trans. Neural Netw..

[10] Lee M., Woo W. ARKB: 3D vision-based Augmented Reality Keyboard. Proceedings of the International Conferece on Artificial Reality and Telexisitence.

[11] Yi X., Yu C., Zhang M., Gao S., Sun K., Shi Y. Atk: Enabling ten-finger freehand typing in air based on 3d hand tracking data. Proceedings of the 28th Annual ACM Symposium on User Interface Software & Technology.

[12] Kim Y.R., Kim G.J. Hovr-type: Smartphone as a typing interface in vr using hovering. Proceedings of the 22nd ACM Conference on Virtual Reality Software and Technology.

[13] Lu Y., Yu C., Yi X., Shi Y., Zhao S. (2017). Blindtype: Eyes-free text entry on handheld touchpad by leveraging thumb’s muscle memory. Proc. ACM Interact. Mob. Wearable Ubiquitous Technol..

[14] Walker J., Kuhl S., Vertanen K. Decoder-assisted typing using an HMD and a physical keyboard. Proceedings of the Extended Abstracts of the the ACM Conference on Human Factors in Computing Systems.

[15] Walker J., Li B., Vertanen K., Kuhl S. Efficient typing on a visually occluded physical keyboard. Proceedings of the 2017 CHI Conference on Human Factors in Computing Systems.

[16] McGill M., Boland D., Murray-Smith R., Brewster S. A dose of reality: Overcoming usability challenges in VR head-mounted displays. Proceedings of the 33rd Annual ACM Conference on Human Factors in Computing Systems.

[17] Knierim P., Schwind V., Feit A.M., Nieuwenhuizen F., Henze N. Physical Keyboards in Virtual Reality: Analysis of Typing Performance and Effects of Avatar Hands. Proceedings of the 2018 CHI Conference on Human Factors in Computing Systems.

[18] Grubert J., Witzani L., Ofek E., Pahud M., Kranz M., Kristensson P.O. Effects of hand representations for typing in virtual reality. Proceedings of the IEEE Conference on Virtual Reality and 3D User Interfaces (VR).

[19] Lin J.W., Han P.H., Lee J.Y., Chen Y.S., Chang T.W., Chen K.W., Hung Y.P. Visualizing the keyboard in virtual reality for enhancing immersive experience. Proceedings of the ACM SIGGRAPH 2017 Posters.

[20] Schick A., Morlock D., Amma C., Schultz T., Stiefelhagen R. Vision-based handwriting recognition for unrestricted text input in mid-air. Proceedings of the 14th ACM international conference on Multimodal interaction.

[21] Gugenheimer J., Dobbelstein D., Winkler C., Haas G., Rukzio E. Facetouch: Enabling touch interaction in display fixed uis for mobile virtual reality. Proceedings of the 29th Annual Symposium on User Interface Software and Technology.

[22] Yu C., Gu Y., Yang Z., Yi X., Luo H., Shi Y. Tap, dwell or gesture?: Exploring head-based text entry techniques for hmds. Proceedings of the 2017 CHI Conference on Human Factors in Computing Systems.

[23] Pratorius M., Burgbacher U., Valkov D., Hinrichs K. (2015). Sensing Thumb-to-Finger Taps for Symbolic Input in VR/AR Environments. IEEE Comput. Graph. Appl..

[24] Sridhar S., Markussen A., Oulasvirta A., Theobalt C., Boring S. Watchsense: On-and above-skin input sensing through a wearable depth sensor. Proceedings of the 2017 CHI Conference on Human Factors in Computing Systems.

[25] Harrison C., Benko H., Wilson A.D. OmniTouch: Wearable multitouch interaction everywhere. Proceedings of the 24th Annual ACM Symposium on User Interface Software and Technology.

[26] Sridhar S., Feit A.M., Theobalt C., Oulasvirta A. Investigating the dexterity of multi-finger input for mid-air text entry. Proceedings of the 33rd Annual ACM Conference on Human Factors in Computing Systems.

[27] Van Vlaenderen W., Brulmans J., Vermeulen J., Schöning J. Watchme: A novel input method combining a smartwatch and bimanual interaction. Proceedings of the 33rd Annual ACM Conference Extended Abstracts on Human Factors in Computing Systems.

[28] Wang S., Song J., Lien J., Poupyrev I., Hilliges O. Interacting with soli: Exploring fine-grained dynamic gesture recognition in the radio-frequency spectrum. Proceedings of the 29th Annual Symposium on User Interface Software and Technology.

[29] Harrison C., Tan D., Morris D. Skinput: Appropriating the body as an input surface. Proceedings of the SIGCHI Conference on Human Factors in Computing Systems.

[30] Chen K.Y., Lyons K., White S., Patel S. uTrack: 3D input using two magnetic sensors. Proceedings of the 26th Annual ACM Symposium on User Interface Software and Technology.

[31] Amma C., Schultz T. Airwriting: Demonstrating mobile text input by 3D-space handwriting. Proceedings of the 2012 ACM International Conference on Intelligent User Interfaces.

[32] Zhang Y., Zhou J., Laput G., Harrison C. Skintrack: Using the body as an electrical waveguide for continuous finger tracking on the skin. Proceedings of the 2016 CHI Conference on Human Factors in Computing Systems.

[33] Kienzle W., Hinckley K. LightRing: Always-available 2D input on any surface. Proceedings of the 27th Annual ACM Symposium on User Interface Software and Technology.

[34] Chen X., Grossman T., Fitzmaurice G. Swipeboard: A text entry technique for ultra-small interfaces that supports novice to expert transitions. Proceedings of the 27th Annual ACM Symposium on User Interface Software and Technology.

[35] Hong J., Heo S., Isokoski P., Lee G. SplitBoard: A simple split soft keyboard for wristwatch-sized touch screens. Proceedings of the 33rd Annual ACM Conference on Human Factors in Computing Systems.

[36] Shibata T., Afergan D., Kong D., Yuksel B.F., MacKenzie I.S., Jacob R.J. DriftBoard: A panning-based text entry technique for ultra-small touchscreens. Proceedings of the 29th Annual Symposium on User Interface Software and Technology.

[37] Oney S., Harrison C., Ogan A., Wiese J. ZoomBoard: A diminutive qwerty soft keyboard using iterative zooming for ultra-small devices. Proceedings of the SIGCHI Conference on Human Factors in Computing Systems.

[38] Yu D., Fan K., Zhang H., Monteiro D., Xu W., Liang H.N. (2018). PizzaText: Text Entry for Virtual Reality Systems Using Dual Thumbsticks. IEEE Trans. Vis. Comput. Graph..

[39] Költringer T., Isokoski P., Grechenig T. TwoStick: Writing with a game controller. Proceedings of the Graphics Interface 2007.

[40] Sandnes F.E., Aubert A. (2006). Bimanual text entry using game controllers: Relying on users’ spatial familiarity with QWERTY. Interact. Comput..

[41] Mithal A.K., Douglas S.A. Differences in movement microstructure of the mouse and the finger-controlled isometric joystick. Proceedings of the SIGCHI Conference on Human Factors in Computing Systems.

[42] Gong J., Xu Z., Guo Q., Seyed T., Chen X., Bi X., Yang X.D. Wristext: One-handed text entry on smartwatch using wrist gestures. Proceedings of the 2018 CHI Conference on Human Factors in Computing Systems.

[43] Isokoski P., Raisamo R. Quikwriting as a multi-device text entry method. Proceedings of the Third Nordic Conference on Human-Computer Interaction.

[44] Cho H., Kim M., Seo K. A text entry technique for wrist-worn watches with tiny touchscreens. Proceedings of the Adjunct Publication of the 27th Annual ACM Symposium on User Interface Software and Technology.

[45] Lyons K., Plaisted D., Starner T. Expert chording text entry on the twiddler one-handed keyboard. Proceedings of the Eighth International Symposium on Wearable Computers.

[46] Rosenberg R., Slater M. (1999). The chording glove: A glove-based text input device. IEEE Trans. Syst. Man Cybern. Part C Appl. Rev..

[47] Way D., Paradiso J. A usability user study concerning free-hand microgesture and wrist-worn sensors. Proceedings of the 2014 11th International Conference on Wearable and Implantable Body Sensor Networks.

[48] Dementyev A., Paradiso J.A. WristFlex: Low-power gesture input with wrist-worn pressure sensors. Proceedings of the 27th Annual ACM Symposium on User Interface Software and Technology.

[49] Thomas B.H., Piekarski W. (2002). Glove based user interaction techniques for augmented reality in an outdoor environment. Virtual Real..

[50] Lehikoinen J., Röykkee M. (2001). N-fingers: A finger-based interaction technique for wearable computers. Interact. Comput..

[51] Bajer B., MacKenzie I.S., Baljko M. Huffman base-4 text entry glove (H4 TEG). Proceedings of the 2012 16th International Symposium on Wearable Computers.

[52] Wong P.C., Zhu K., Fu H. FingerT9: Leveraging thumb-to-finger interaction for same-side-hand text entry on smartwatches. Proceedings of the 2018 CHI Conference on Human Factors in Computing Systems.

[53] Whitmire E., Jain M., Jain D., Nelson G., Karkar R., Patel S., Goel M. (2017). Digitouch: Reconfigurable thumb-to-finger input and text entry on head-mounted displays. Proc. ACM Interact. Mob. Wearable Ubiquitous Technol..

[54] Jiang H., Weng D., Zhang Z., Bao Y., Jia Y., Nie M. HiKeyb: High-Efficiency Mixed Reality System for Text Entry. Proceedings of the 2018 IEEE International Symposium on Mixed and Augmented Reality Adjunct (ISMAR-Adjunct).

[55] Budhiraja P., Sodhi R., Jones B., Karsch K., Bailey B., Forsyth D. (2015). Where’s my drink? Enabling peripheral real world interactions while using HMDs. arXiv.

[56] MacKenzie I.S. (1992). Fitts’ law as a research and design tool in human-computer interaction. Hum.-Comput. Interact..

[57] MacKenzie I.S. KSPC (keystrokes per character) as a characteristic of text entry techniques. Proceedings of the International Conference on Mobile Human-Computer Interaction.

[58] Gopher D., Raij D. (1988). Typing with a two-hand chord keyboard: Will the qwerty become obsolete?. IEEE Trans. Syst. Man Cybern..

[59] MacKenzie I.S., Zhang S.X. The design and evaluation of a high-performance soft keyboard. Proceedings of the SIGCHI Conference on Human Factors in Computing Systems.

[60] Barrett J., Krueger H. (1994). Performance effects of reduced proprioceptive feedback on touch typists and casual users in a typing task. Behav. Inf. Technol..

[61] Hart S.G., Staveland L.E. (1988). Development of NASA-TLX (Task Load Index): Results of empirical and theoretical research. Advances in Psychology.

[62] Gentner D.R., Grudin J.T., Larochelle S., Norman D.A., Rumelhart D.E.A., Rumelhart D.E. (1983). A glossary of terms including a classification of typing errors. Cognitive Aspects of Skilled Typewriting.

